# Metastatic Lung Cancer to the Distal Finger Presenting as Osteomyelitis

**DOI:** 10.7759/cureus.11441

**Published:** 2020-11-11

**Authors:** Liza Garabet Diramerian, Edward Griffin, Kenneth Pendergrast, Edward Arsura, Marigny Roberts

**Affiliations:** 1 Internal Medicine, Kaweah Delta Health Care District, Visalia, USA; 2 Internal Medicine, LewisGale Medical Center, Salem, USA; 3 Internal Medicine, Salem Veterans Affairs Medical Center, Salem, USA; 4 Pathology, LewisGale Medical Center, Salem, USA

**Keywords:** osteomyelitis, micro metastasis, trauma, epidermal growth factor

## Abstract

Metastasis to distal phalanx is a rare site for metastasis. It is often misdiagnosed as osteomyelitis because of similar clinical features, symptoms, and radiologic findings. If preceded by trauma, the diagnosis could be difficult. We are presenting a case of a 69-year-old male cigarette smoker, who presented with progressive painful swelling of the right second digit for two months duration after he lacerated his finger by a fingernail clipper. After receiving several unsuccessful courses of antibiotics, he was admitted for further treatment. Based on the CT scan of the right hand, he was treated for osteomyelitis and scheduled for elective surgery. As a part of the preoperative workup, his chest X-ray (CXR) revealed a left lower lobe infiltrate, and a subsequent CT of the chest demonstrated a 6 cm mass in the left lower lobe. The pathologic findings of lung mass and finger biopsy revealed a poorly differentiated carcinoma. The patient was treated with several cycles of chemotherapy before he decided to seek hospice care.

Certain malignancies have increased receptors for wound-healing factors. For those malignancies, trauma will promote local metastasis by releasing wound-healing factors that create a favorable environment for micrometastasis cell growth.

Some of these components currently are targets for therapy, while other components may be targets for therapy in the future.

## Introduction

The distal phalanx is a rare site of metastatic disease and is associated with a poor prognosis. The lung is the most common primary site of origin for acrometastases (40%-50%) [1]. Some 0.2% of lung cancer present with acral metastasis [2-3]. Distal phalanx metastasis is frequently misdiagnosed as osteomyelitis because of similar clinical features and radiologic findings. If preceded by trauma, the diagnosis may be particularly difficult. We present a patient who lacerated his distal finger and had a lesion that was initially diagnosed as a superficial skin infection that progressed to osteomyelitis but was subsequently found to be a metastatic lesion. We also present a proposed mechanism for this unusual site of metastasis.

There are only a few cases with similar presentations reported in the literature, but we were unable to find any with a definite history of antecedent trauma.

## Case presentation

**Figure 1:**
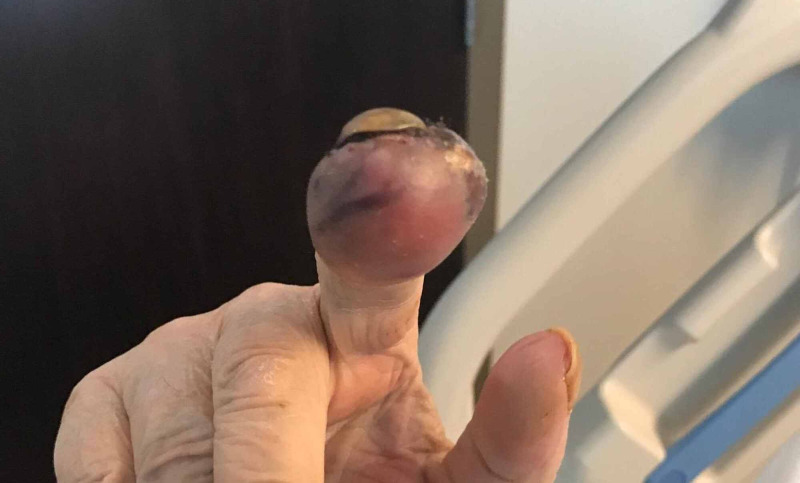
Picture of the involved hand at the time of admission.

**Figure 2:**
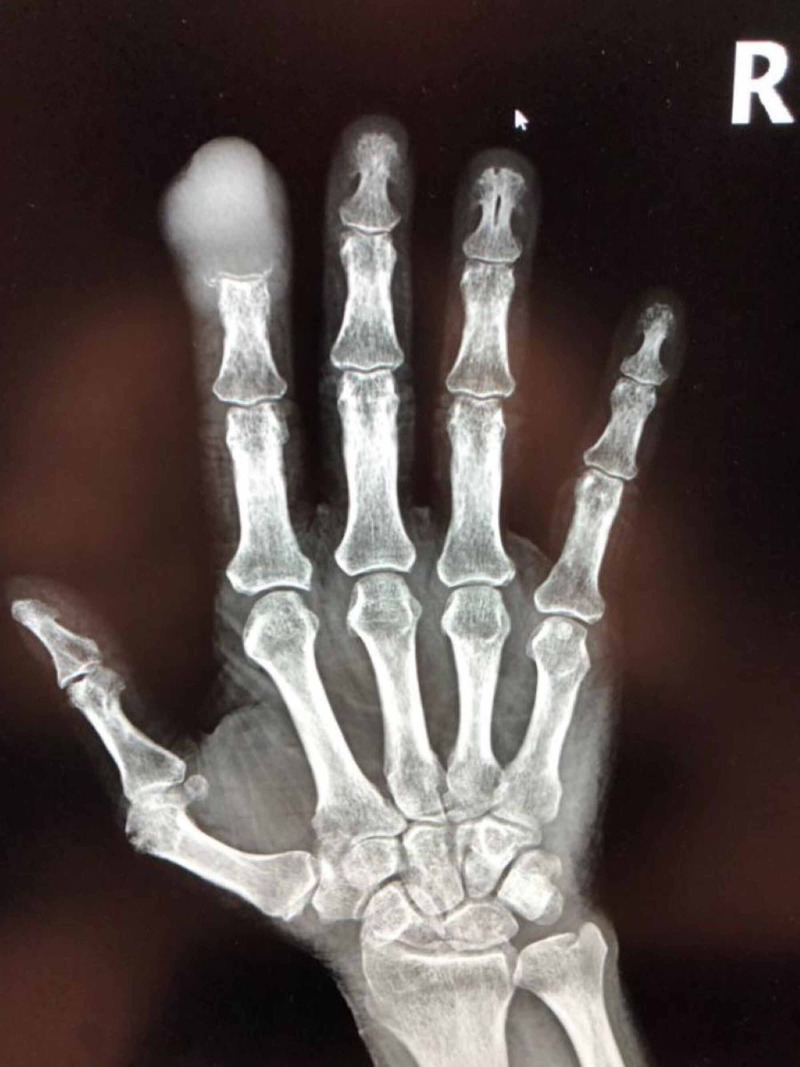
Plain X-Ray of the involved hand taken at admission.

The patient is a 69-year-old male cigarette smoker who presented with complaints of pain in the right second digit of two months duration. It was associated with erythema and marked edema. The symptoms developed shortly after the patient lacerated his finger while trimming his fingernails. The pain was throbbing in nature, did not radiate, and there was no aggravating or alleviating factors. He denied fever, chills, and sweats. Over a period of a month after the initial laceration, the lesion gradually enlarged and became more painful. He sought care from his primary care provider who prescribed trimethoprim/sulfamethoxazole for presumed infection. He failed to improve. A week later he presented to a local hospital ER where plain films were read as being consistent with osteomyelitis. He was discharged on cephalexin and plans made for elective surgery. Approximately a week later the involved finger began hemorrhaging and he presented to our institution. The distal phalanx was markedly inflamed, tender, and showed signs of recent hemorrhage (Figure 1). There was no lymphadenopathy. X-Rays (Figure 2) of the digit at this time revealed erosion of the second distal phalanx felt to be consistent with osteomyelitis. He was admitted for definitive care with an initial diagnosis of osteomyelitis. His past medical history was notable solely for remote accidental brain trauma with retained metal fragments in the brain.

**Figure 3:**
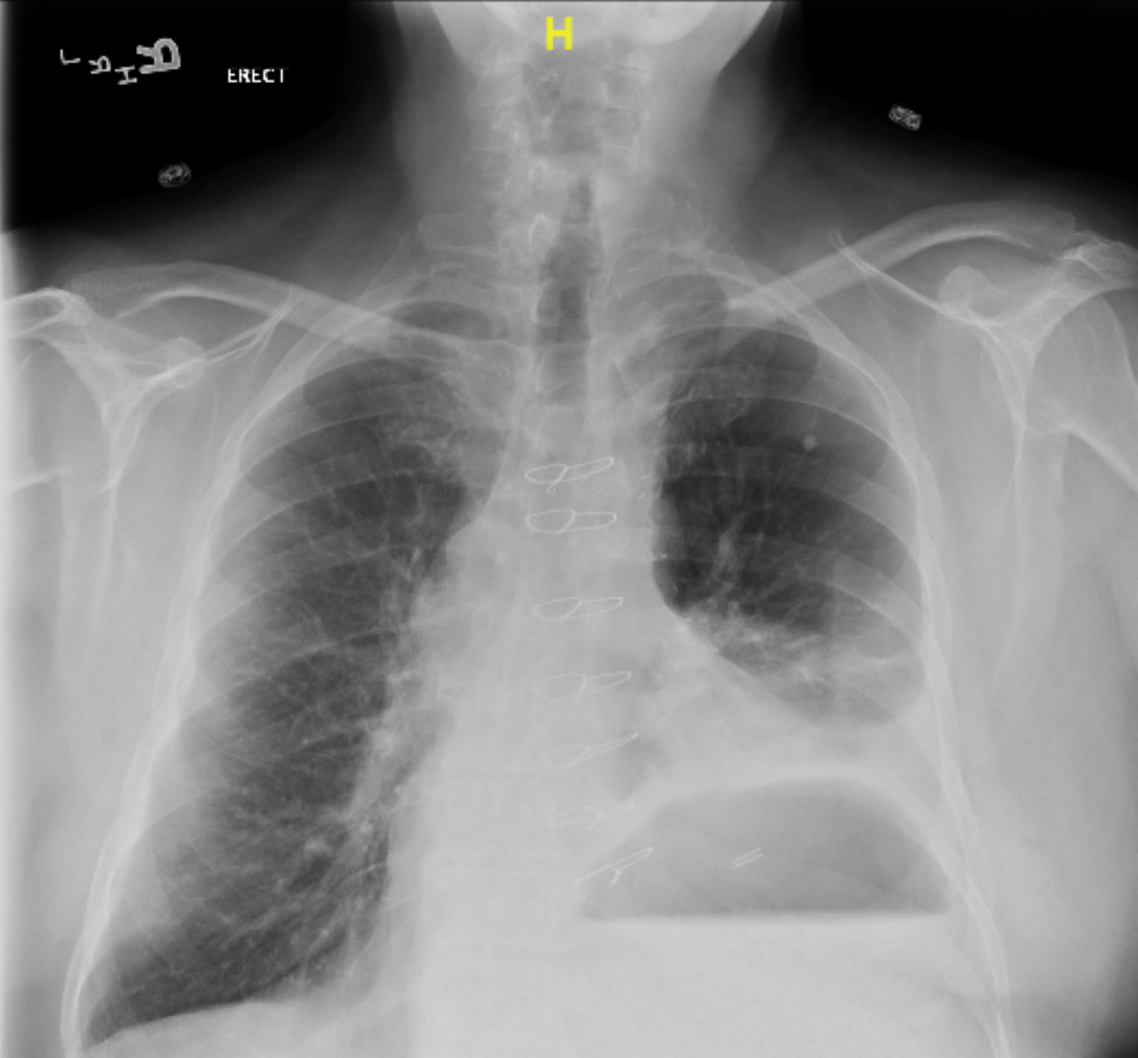
A CXR revealed a left lower lobe infiltrate. CXR, chest X-ray

**Figure 4:**
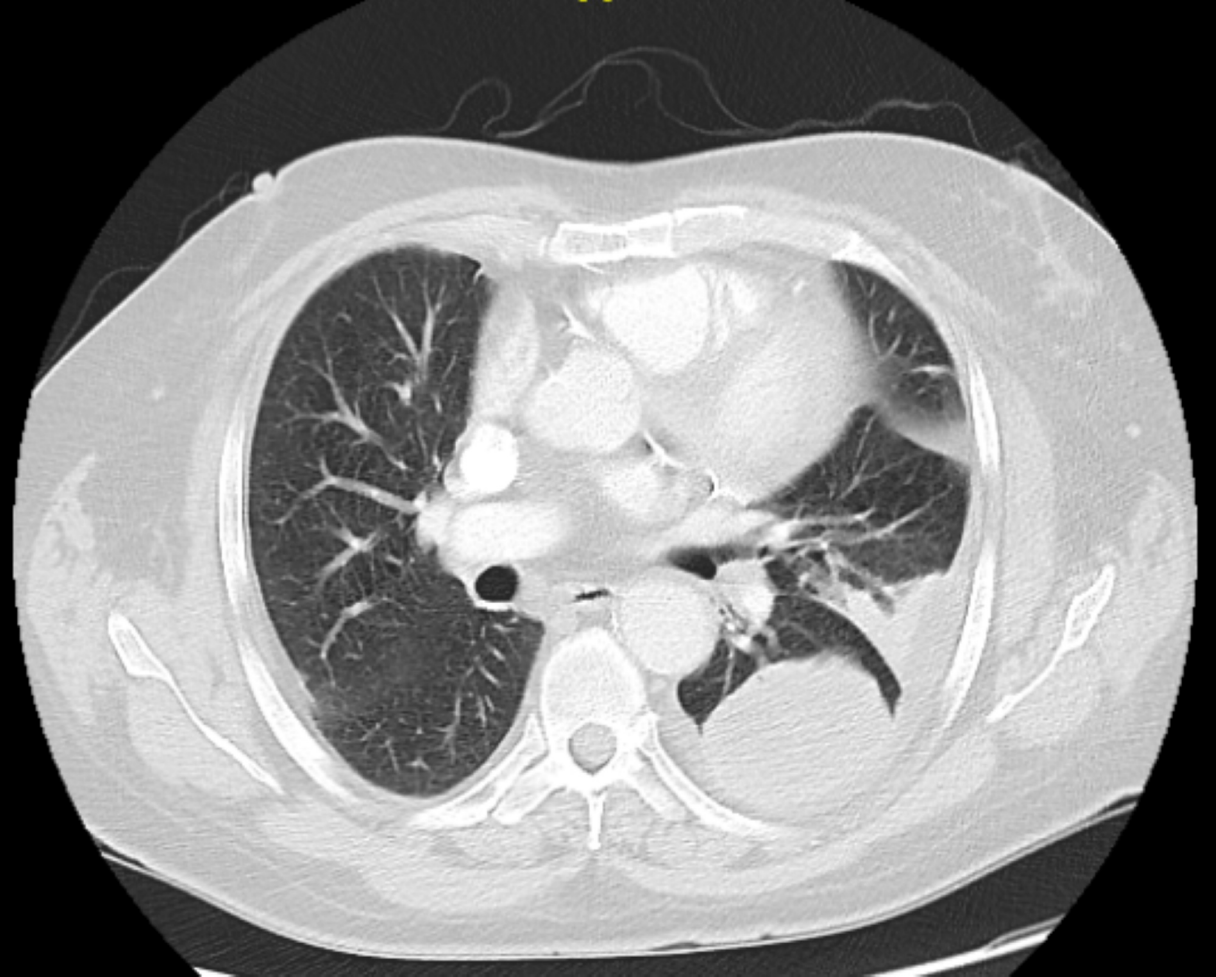
CT scan of the chest with and without contrast, shows a large round mass-like lesion measuring 6 cm in the left lower lobe of the lung without evidence of nodal involvement or metastatic disease.

 Laboratory exam was significant for an erythrocyte sedimentation rate (ESR) of 26, and normal white blood cell (WBC). CT scan of the right hand was interpreted as an infectious process with the destruction of distal phalanx suggestive of severe osteomyelitis. As a part of perioperative workup, a chest X-ray (CXR) revealed a left lower lobe infiltrate (Figure 3). However, as he had no signs or symptoms of pneumonia, a CT scan of the chest was obtained and it demonstrated a large, round mass-like lesion measuring 6 cm in the left lower lobe of the lung without evidence of nodal involvement or metastatic disease (Figure 4).

**Figure 5:**
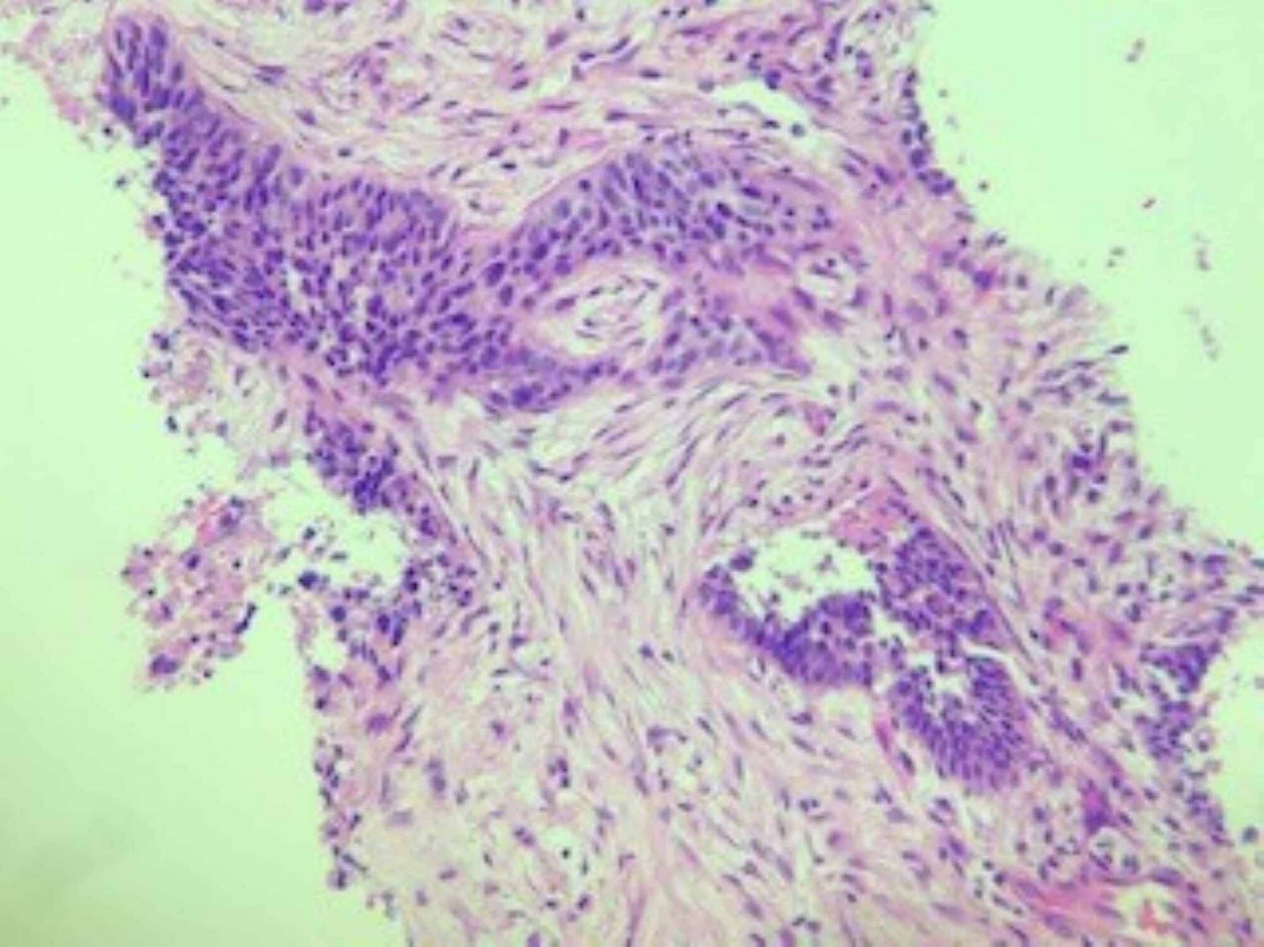
Biopsy of left lower lobe lung mass shows a poorly differentiated, nested tumor comprised of malignant epithelial cells with focal basaloid features. The background is a desmoplastic and inflamed stroma.

**Figure 6:**
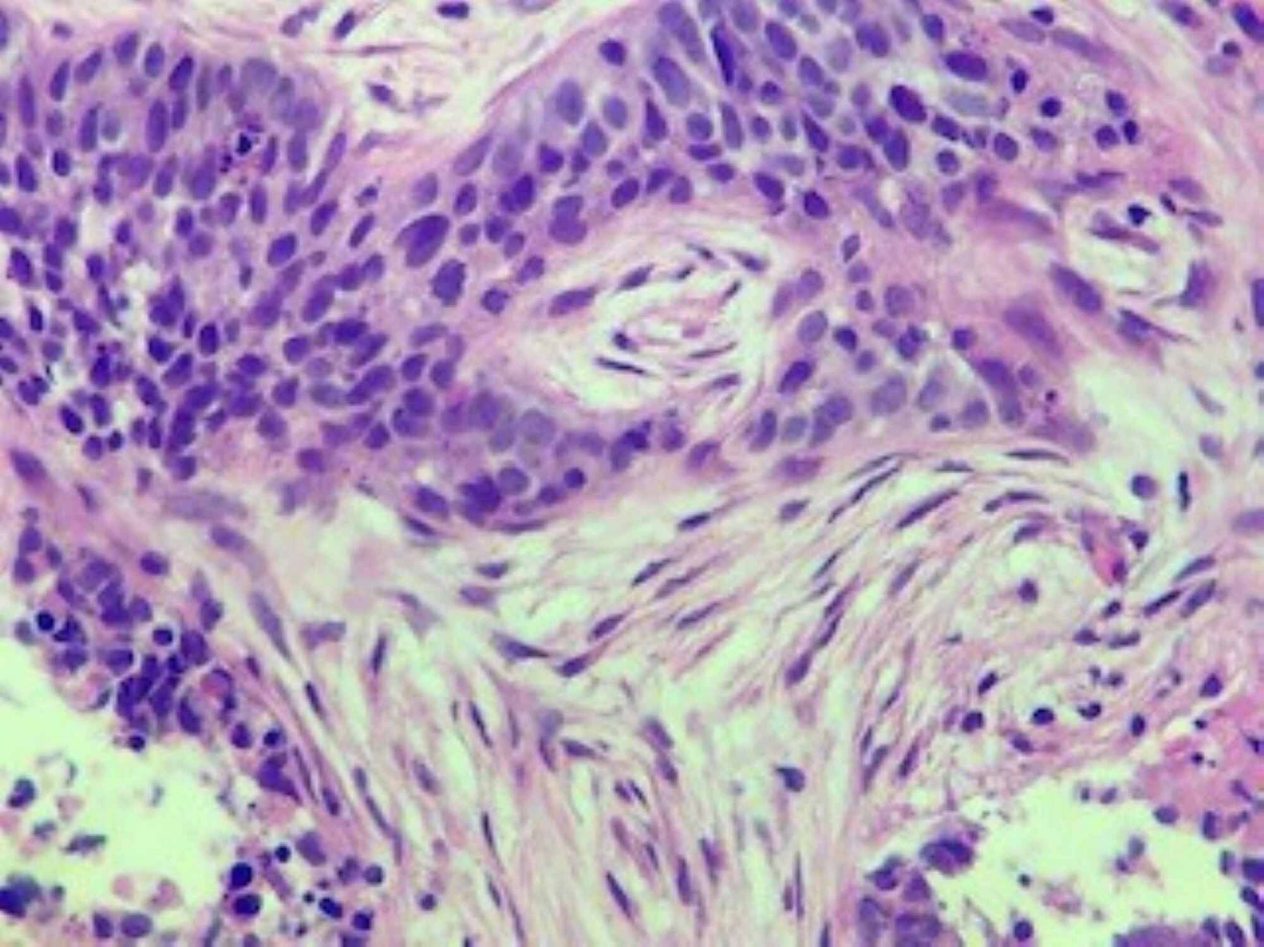
Higher power view of the lung tumor demonstrating areas of tumor necrosis (bottom left and right).

**Figure 7:**
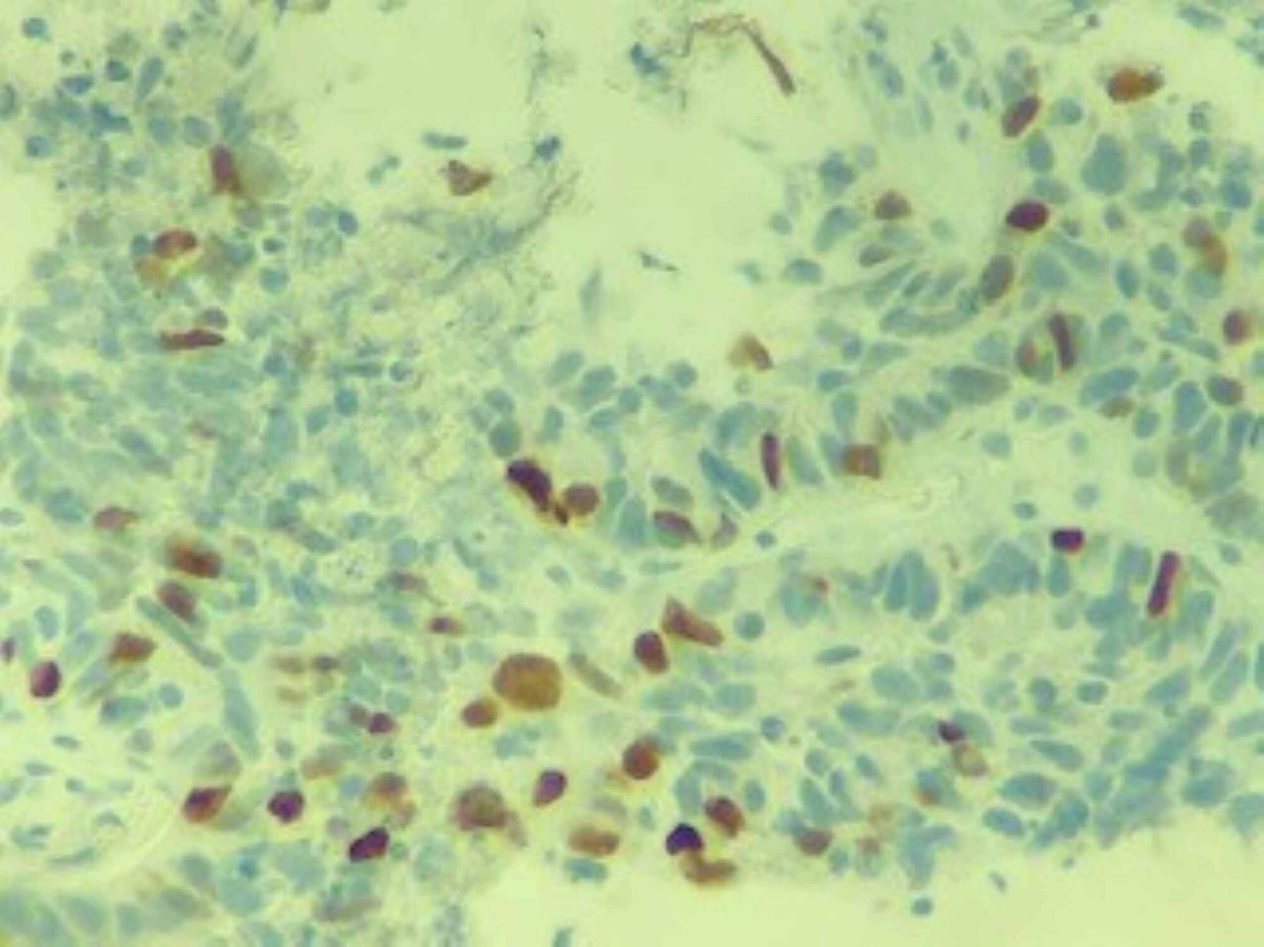
Immunoreactivity in the tumor cells with p63, a marker specific for squamous cell carcinoma.

**Figure 8:**
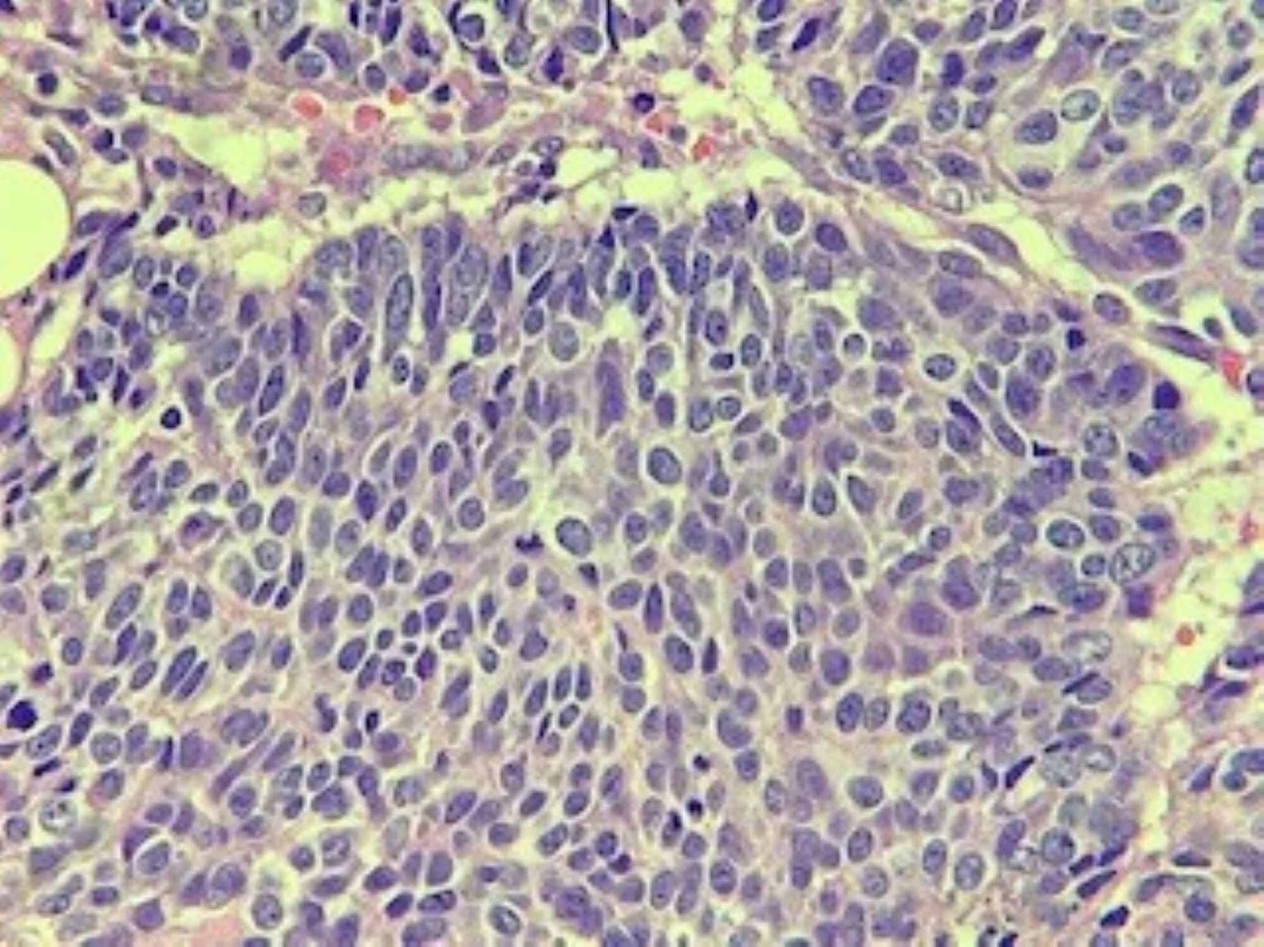
High power view of the tumor in the right index finger. Tumor is poorly differentiated with a basaloid morphology.

**Figure 9:**
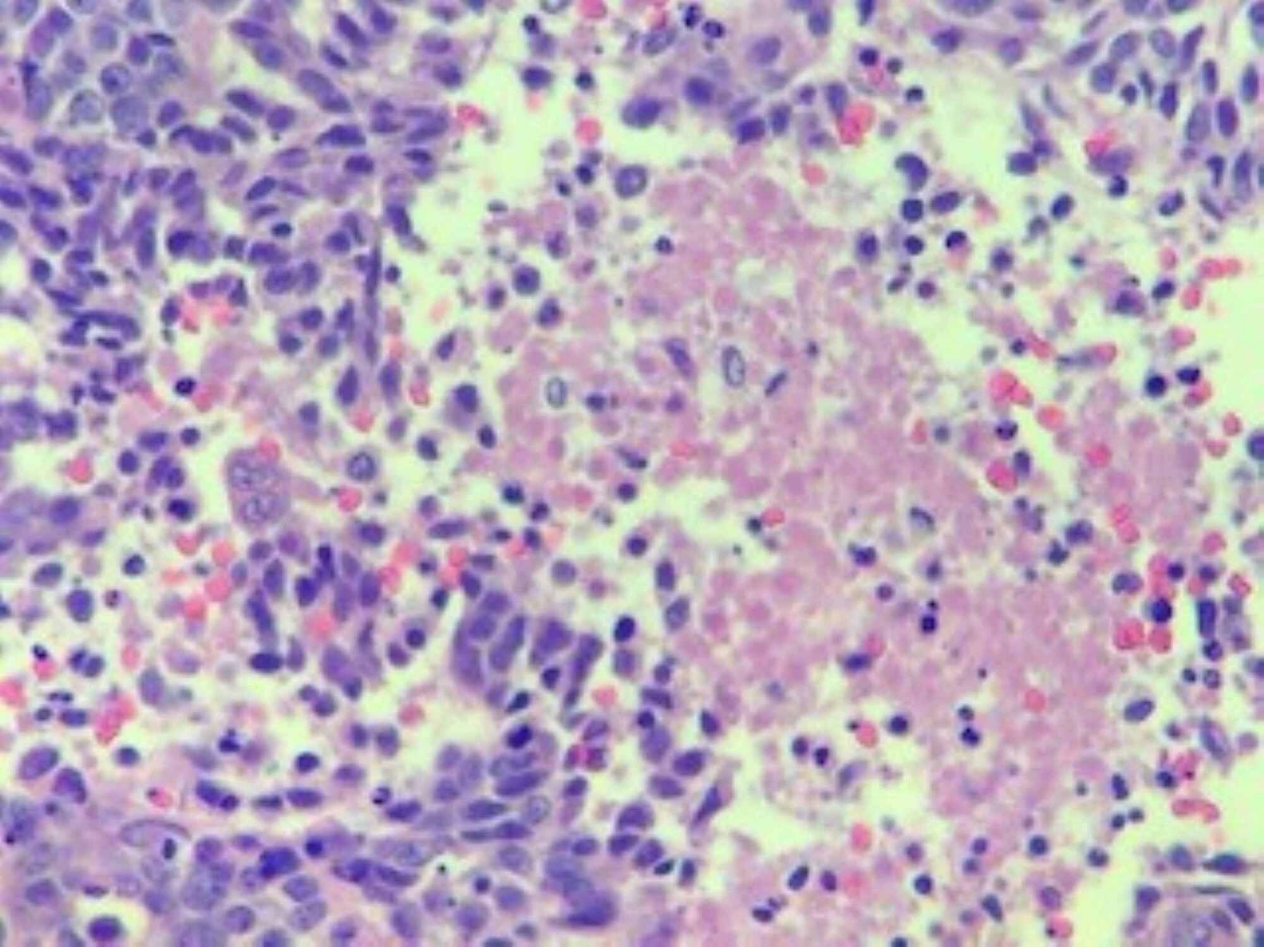
Right index finger tumor with central necrosis and apoptotic tumor debris.

**Figure 10:**
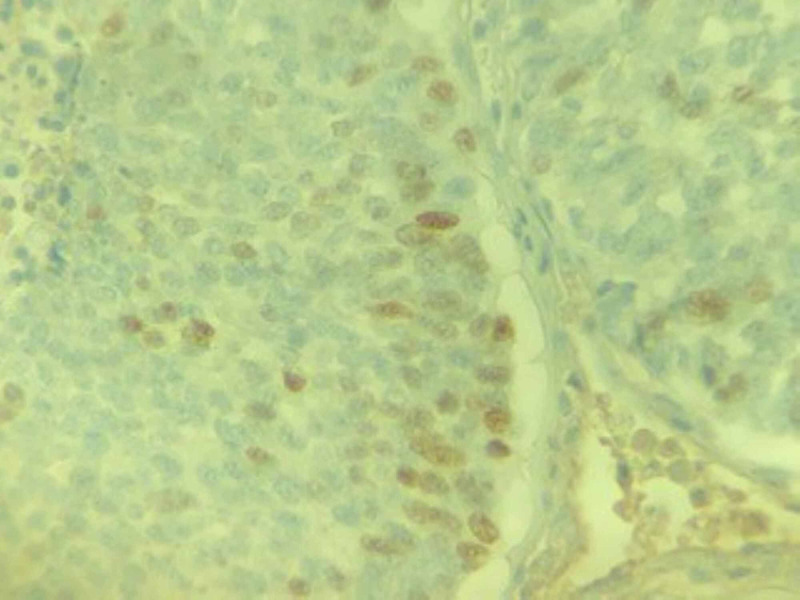
Right index finger tumor demonstrating nuclear immunoreactivity with p63, a specific marker for squamous cell carcinoma.

A CT-guided biopsy of the lung lesion revealed poorly differentiated carcinoma with extensive necrosis. Patchy p63 staining was suggestive of a diagnosis of poorly differentiated squamous cell carcinoma (Figures 5-7). A biopsy of the distal phalangeal lesion was consistent with that of lung cancer (Figures 8-10).

The CT scan of the brain was done in lieu of an MRI because of the presence of metallic fragments and it revealed two lesions, one in the right cerebellum measuring approximately 1.1 cm x 1.1 cm and a left periatrial 4 mm x 5 mm lesion. His case was discussed in a multidisciplinary cancer conference and chemotherapy and radiation to the chest with a reassessment of the brain following completion of the cycle of radiation and chemotherapy. After several cycles of chemotherapy, the patient decided to stop therapy and seek hospice care.

## Discussion

Both experimental [4] and clinical evidence [5] support the hypothesis that antecedent trauma facilitates the development of metastases of cancer to the site of the trauma. Sundaram and colleagues noted the wound-healing process can be “hijacked” by tumors to promote invasion and metastases [6]. It is well known that the amount of circulating tumor cells is important in disease progression and metastasis. It is likely that the wound healing process at the site of trauma promotes metastasis at the trauma site. Part of the wound-healing process involves increased local levels of epidermal growth factor (EGF) and factor follistatin-like 1 (FSTL1) [6].

Acrometastases are rare, representing only 0.1% of all skeletal metastases [7]. The lung is the most common primary site of origin for acrometastases (40%-50%) [1]. Squamous cell cancer of the lung is the most common type of histology, as in the present case [8]. Squamous cell cancer of the lung is rich in epidermal growth factor receptors (EGFR) with a 2.5-fold amount than that of normal skin. The EGF levels and other factors are increased at the site of a wound [9].

The mechanisms behind acrometastases are not well defined. We propose that trauma and even microtrauma may contribute to the pathogenesis of acrometastases, as recent literature shows the dominant hand more frequently developed acrometastases [10]. The increase in epidermal growth factor in particular and, likely other factors related to wound healing result in an increased likelihood that circulating tumor cells will form a macro-metastatic lesion at the site of the wound, particularly in the case of malignant cells harboring increased numbers of EGFR, as is the case with squamous cell carcinoma of the lung. Other wound-healing factors are likely involved as well. 

Aberrant activation of FSLT1 and downregulation of miRvia EGF-dependent suppression of KSRP - part of the wound-healing process, act as drivers of metastasis in head and neck squamous cell cancer [6].

FSLT1 promotes the expression of matrix metalloprotein MMP9, which can degrade the extracellular matrix through the effect on mitogen-activated protein kinase (MAP kinase) signaling, so this will enhance the likelihood of metastasis to that area. Additionally, Sundaram and colleagues (2017) [6] found that the miR -198 target DIAPH, which is known to promote migration is increased in this setting. 

Many of these components and others may be future targets for therapy for the prevention or treatment of metastases [6, 11-12].

It is important for physicians to recognize that a persistent lesion at the site of a wound with osteomyelitis presentation of the finger may be due to underlying malignancy. This recognition will improve patient care, may lead to earlier diagnosis of malignancy, and may prevent unnecessary procedures.

## Conclusions

We present this case to highlight that physicians should consider a metastatic lesion in a patient with a no resolving infection, even when there is associated trauma that appears to have been the precipitant. This should be especially considered in patients with risk factors for lung cancer, such as smoking. This recognition will improve patient care, may lead to earlier diagnosis of malignancy, and prevent unnecessary surgery.

We also would like to highlight that there is increasing evidence that trauma promotes metastasis and that the wound healing process is important in promoting metastasis at the site of the primary tumor and we suspect, by promoting local adherence and growth of circulating tumor cells.
